# What primary health care services should residents of rural and remote Australia be able to access? A systematic review of “core” primary health care services

**DOI:** 10.1186/1472-6963-13-178

**Published:** 2013-05-17

**Authors:** Timothy A Carey, John Wakerman, John S Humphreys, Penny Buykx, Melissa Lindeman

**Affiliations:** 1 Centre for Remote Health, Flinders University and Charles Darwin University, Alice Springs, Australia; 2 Centre of Research Excellence in Rural and Remote Primary Health Care, Bendigo, Australia; 3 Central Australian Mental Health Service, Alice Springs, Australia; 4 School of Rural Health, Monash University, Clayton, Australia

**Keywords:** Primary health care, Core services, Access, Equity, Rural and remote

## Abstract

**Background:**

There are significant health status inequalities in Australia between those people living in rural and remote locations and people living in metropolitan centres. Since almost ninety percent of the population use some form of primary health care service annually, a logical initial step in reducing the disparity in health status is to improve access to health care by specifying those primary health care services that should be considered as “core” and therefore readily available to all Australians regardless of where they live. A systematic review was undertaken to define these “core” services.

Using the question “What primary health care services should residents of rural and remote Australia be able to access?”, the objective of this paper is to delineate those primary health care core services that should be readily available to all regardless of geography.

**Method:**

A systematic review of peer-reviewed literature from established databases was undertaken. Relevant websites were also searched for grey literature. Key informants were accessed to identify other relevant reference material. All papers were assessed by at least two assessors according to agreed inclusion criteria.

**Results:**

Data were extracted from 19 papers (7 papers from the peer-reviewed database search and 12 from other grey sources) which met the inclusion criteria. The 19 papers demonstrated substantial variability in both the number and nature of core services. Given this variation, the specification or synthesis of a universal set of core services proved to be a complex and arguably contentious task. Nonetheless, the different primary health care dimensions that should be met through the provision of core services were developed. In addition, the process of identifying core services provided important insights about the need to deliver these services in ways that are “fit-for–purpose” in widely differing geographic contexts.

**Conclusions:**

Defining a suite of core primary health care services is a difficult process. Such a suite should be fit-for-purpose, relevant to the context, and its development should be methodologically clear, appropriate, and evidence-based. The value of identifying core PHC services to both consumers and providers for service planning and monitoring and consequent health outcomes is paramount.

## Background

The implementation of primary health care (PHC) may well be one of the most significant systemic and ideological health reforms of modern times [1]. Countries with stronger PHC systems have demonstrably more efficient, effective, and equitable health care [2]. Primary health care can be considered a philosophy, an approach to the delivery and development of services and first contact health services. It is based on a social, rather than biomedical, model of health, with accessibility to and affordability of services as primary objectives [3]. Issues of accessibility and affordability are particularly relevant in rural and remote Australia where health outcomes are generally worse than those of metropolitan areas and where these significant and unacceptable health inequities persist despite increases in health funding and changes to government policies in recent years [4].

If we are to improve access to PHC services, it is important to identify the nature of these first contact services. In other words, which PHC services might be considered essential or “core” within a health system, and, therefore, readily available at times of need? In the absence of any available listing or register of such services, a systematic review was undertaken with the objective of answering the question “What primary health care services should residents of rural and remote Australia be able to access?”

### Why is it important to define core PHC services?

Since PHC services are the first point of contact to the health system at times of need for most people, their availability is a critical factor determining health care utilisation. Defining those PHC services considered to be “core” is vitally important in order to evaluate their contribution to a reduction in health inequalities, especially those resulting from demographic and geographical factors such as population isolation, dispersed settlement pattern, and the higher proportion of Indigenous citizens in more remote areas in large countries such as Australia and Canada. Cumming [5] cites a number of reasons for defining core services, including the need to:

• Delimit a minimum package of services;

• Inform consumers what they can expect to access;

• Reduce variation in services across geographical areas;

• Prioritise the most important services; and

• Promote competition and efficiency in a market-driven system through comparison of health services provision.

At the same time, however, there may be political and economic reasons as to why policymakers would *not* want to commit to a defined set of core PHC services. Professor Martin Roland, for example, confirms that core services have deliberately never been defined in the United Kingdom (personal communication, 9 February 2012) and Associate Professor Jackie Cumming reported that the Core Services Committee in New Zealand (which became the National Health Committee) also decided not to determine a set of core services (personal communication, 28 February 2012). Defining core PHC services implies a financial and ethical responsibility to ensure that these services are readily accessible for all people in a health system.

Given Australia’s vast and diverse geography and settlement pattern, issues of access to health care services have been long-standing and most significant for people living in remote and rural areas. The difficulty in ensuring adequate access to health services in such communities where “market-based” provision does not result in the equitable provision of health care is one strong reason why defining a set of core PHC services is so important. Moreover, given that the nature and priority accorded to health needs may differ between geographical areas, it is important that people at all levels of responsibility within a health system are very clear about the services that the health system is obligated to provide in order to ensure equitable access to appropriate health care at times of need. Impartial discussions about the resourcing implications (financial and otherwise) of providing services to all within a given health system can only proceed *after* a set of core services have been defined.

The provision of PHC services is an international issue spanning both developed and developing countries. To that end, a systematic review of international literature was undertaken in order to identify “core” primary health care services. Defining “core” services is a pre-requisite to examining differences in access to PHC, and can only proceed once some delimitation of the set of core services is available. In short, knowing which primary health care services can be considered essential or “core”, will help overcome a major knowledge gap for health service planners concerned to overcome access and equity disparities in the provision of health services.

## Methods

### Search strategy

Preliminary scoping searches of established databases such as Medline and EBSCO were implemented. A librarian assisted with the development of the search strategy. Inclusion and exclusion criteria are documented in Table 1. (A review protocol was not established in the public domain; however, procedures and forms are available from the corresponding author).

**Table 1:** Inclusion and exclusion standards according to specified criteria

**Criteria**	**Inclusion**	**Exclusion**
Time period	All	
Language	English	
Geographical delimitation	All countries	
Level of Development	All	
Level of health care	PHC services	Secondary, tertiary services
Aim: to identify the services that should be considered “core” to a rural and remote Australian primary health care service.	• Identifies range, package, or suite of PHC services	• Identifies only one or a small number of disease specific or organ specific services
	• Relevant to Australian rural and remote context	• Not relevant to Australian rural and remote context
Methods	• Single or multiple methods documented	• No explicit method described

Initial searches indicated that it would be prudent to search broadly using a variety of terms. A number of large, established, peer-reviewed literature databases were searched. “Grey” literature was accessed through websites and key contacts. Table 2 lists the databases and websites that were searched. In general, the same terms that were used for the database searches were used when searching websites for the grey literature, although “primary care” was used rather than “primary health care” because it was a more effective term in returning hits on the websites.

**Table 2:** Searches conducted throughout databases and the grey literature

**Type of Information**	**Source**	**Strategy**
Databases	• Ovid Medline	“Primary Health Care/”; essential adj4 service$; core adj4 service$; medical necessity; basic adj4 service$; comprehensive adj4 service$; community access; acceptable adj4 service$; minimum adj4 service$; fundamental adj4 service$; element$ adj4 service$
	• Ebsco CINAHL	
	• Informit	
	• Cochrane Library	
Grey Literature	• Institute of Medicine (http://www.iom.edu/Reports.aspx)	The same search terms were used for these websites as was used for the databases.
	• Healthinfonet (http://www.healthinfonet.ecu.edu.au/)	
	• Lowitja Institute (http://www.lowitja.org.au/)	
	• Primary Care Partnerships in Victoria (http://www.health.vic.gov.au/pcps/about/index.htm)	
	• American College of Physicians (http://www.acponline.org/)	
	• Bureau of Health Professions (http://bhpr.hrsa.gov/)	
	• Health Systems Evidence (http://www.healthsystemsevidence.org/)	

After papers were selected from the databases, reference lists of these papers were perused in a “snowballing” process to obtain other potential papers. Key informants, including members of the research team, also provided papers from their own resources (such as contact with primary health care services).

### Methods of screening and selection criteria

**Figure 1:**
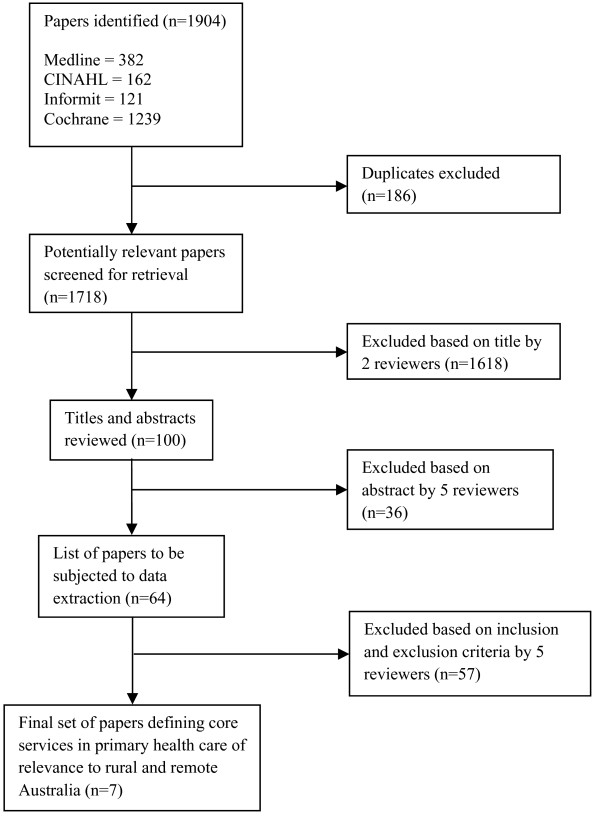
Electronic database selection process.

Figure 1 describes schematically the screening process of selecting papers from the database. After removing duplicate titles, two reviewers scanned the titles from the total pool of titles obtained from the search. Then the research team reviewed titles and abstracts to select the most suitable papers according to the research question and the inclusion and exclusion selection criteria (Table 1). As specified in Table 1, the selection criteria included variables such as the time period and the geographical distribution of the papers. The full papers from this set were collected and the same team of reviewers used a standard data extraction pro-forma to further refine the selected sample. To complete this task, four of the researchers worked in two pairs, with each pair reviewing half of the selected set. Where there were discrepancies within the pair, these were first discussed by the pair and, if consensus could not be reached, a fifth researcher adjudicated (Table 2).

### Data extraction

With the final set of papers selected, the primary interest was the particular suite of core services documented, and the methods used to derive these services. The data extraction process therefore included information about the specific services as well as the methods employed. Quality was also assessed using criteria based on questions such as “Is this study underpinned by a strong body of knowledge?” and “Does the method accord with the objectives of the study?”. Other contextual information relevant to our research question was also noted, such as the geographic focus of the paper and the year of the study, in order to assist the transferability and currency of the research findings (Figure 1).

## Results

### Study characteristics

Figure 1 describes the process of screening the initial 1904 papers selected from the electronic data bases down to seven which were finally included for data extraction. From the websites, a further 787 titles were initially obtained, from which only five full papers were ultimately reviewed from these titles. However, none of these papers met selection criteria. Snowballing resulted in an additional six papers that met the inclusion criteria. Key informants identified six papers. Table 3 summarises author details, how the papers were located, and the actual number of core services specified in each of the 19 papers.

**Table 3:** Final set of papers including how they were obtained and the number of core services specified

**Author/s and year**	**How located**	**Number of core services specified**
Asuzu & Ogundeji [6]	Snowballed	10
APHCRI [7]	Snowballed	5
Bartlett & Boffa [8]	Electronic black database	4
Bobadilla et al. [9]	Snowballed	11
Brener et al. [10]	Electronic black database	42
CRH [11]	Key Informant	23
Farrow et al. [12]	Electronic black database	31 (in 3 clusters)
AH&MRC [13]	Electronic black database	101 (in 8 clusters)
OATSIH [14]	Electronic black database	4
Lyle & Kerr [15]	Key Informant	12
McDonald [16]	Electronic black database	16
NACCHO [17]	Snowballed	101 (in 8 clusters)
NATSIHC [18]	Electronic black database	6
Scrimgeour [19]	Key Informant	4 (Canada),
		6 (Australia)
Scrimgeour [20]	Key Informant	4 (Health Transfer Policy Canada)
		14 (Montreal Lake Canada)
		6 (Prince Albert Grand Council Transfer Agreement)
Tilton & Thomas [21]	Key Informant	25
WACHS [22]	Key Informant	11
World Bank [23]	Snowballed	11
WHO [24]	Snowballed	8

### Primary health care core services

Table 3 demonstrates the marked variability in the number and the categorisation of core PHC services. This reflects, at least in part, the differences in terminology used. In some papers e.g., [17] the terms “service” and “function” were used interchangeably, and activities as diverse as “Otitis Media examination and testing” (p. 55) and “Prison advocacy services” (p. 57) were included. In another paper [6] minimum standards were specified and these services included “Provision of HIV counselling and testing centers and services” (p. 85) as well as “Pest control services” (p. 83). In a third paper [15] core activities included “antenatal care” (p. 159) as well as “evaluation of activities” (p. 159).

Some papers specified *what* primary health care services should be provided as well as *how* they should be organized in order to best deliver these services. The “how” included service elements such as infrastructure, staff training, and IT support.

### Data synthesis

The heterogeneous nature of the different sets of core services was due in part to different methods used to derive the sets of services. The different approaches resulted nevertheless in a number of common dimensions used to define the services. These dimensions are summarised in Table 4, as well as the way in which these dimensions were demarcated.

**Table 4:** Dimensions and demarcations upon which to base a set of core services

**Dimensions**	**Demarcations**
1. Function of service	1.1 Getting people better
	1.2 Keeping people well
2. Gender	2.1 Male
	2.2 Female
3. Life Span	3.1 Pre natal
	3.2 Birth
	3.3 Baby
	3.4 Child
	3.5 Youth
	3.6 Adult
	3.7 Elderly (including end of life)
4. Target of service	4.1 Individual
	4.2 Group (including couple, family, and community)
5. Type of Presentation	5.1 Acute
	5.2 Chronic
6. Aspect of Body	6.1 Physical
	6.2 Mental
	6.3 Dental

Due to the variability of results in the 19 papers reviewed, it was not possible to delineate a universal or definitive set of core services. As an example, one set of core services was selected to assess the usefulness of the dimensions and demarcations that were synthesised. Table 5 illustrates how the eight core services specified at the Alma-Ata conference [24] address the demarcations specified in Table 4.

**Table 5:** **Suite of primary health care core services specified at the Alma-Ata conference**[23]**and the demarcations they address**

**Core service**	**Demarcations addressed**																	
	**1.1**	**1.2**	**2.1**	**2.2**	**3.1**	**3.2**	**3.3**	**3.4**	**3.5**	**3.6**	**3.7**	**4.1**	**4.2**	**5.1**	**5.2**	**6.1**	**6.2**	**6.3**
1. Education concerning prevailing health problems and the methods of preventing and controlling them	**X**	**X**	**X**	**X**	**X**	**X**	**X**	**X**	**X**	**X**	**X**	**X**	**X**	**X**	**X**	**X**	**X**	**X**
2. Promotion of food supply and proper nutrition		**X**	**X**	**X**	**X**	**X**	**X**	**X**	**X**	**X**	**X**		**X**	**X**	**X**	**X**	**X**	**X**
3. An adequate supply of safe water and basic sanitation	**X**	**X**	**X**	**X**	**X**	**X**	**X**	**X**	**X**	**X**	**X**		**X**	**X**	**X**	**X**	**X**	**X**
4. Maternal and child health care, including family planning	**X**	**X**	**X**	**X**	**X**	**X**	**X**	**X**		**X**		**X**	**X**	**X**	**X**	**X**	**X**	**X**
5. Immunization against the major infectious diseases		**X**	**X**	**X**		**X**	**X**	**X**	**X**	**X**	**X**		**X**			**X**	**X**	
6. Prevention and control of locally endemic diseases		**X**	**X**	**X**	**X**	**X**	**X**	**X**	**X**	**X**	**X**		**X**	**X**	**X**	**X**		
7. Appropriate treatment of common diseases and injuries	**X**		**X**	**X**			**X**	**X**	**X**	**X**	**X**	**X**		**X**		**X**		**X**
8. Provision of essential drugs	**X**	**X**	**X**	**X**	**X**	**X**	**X**	**X**	**X**	**X**	**X**	**X**		**X**	**X**	**X**	**X**	**X**

## Discussion

The heterogeneity of the papers reviewed in this study precluded the description or synthesis of a definitive or universal set of core PHC services thus limiting our ability to answer our original research question. The variability in published material was a result of the different purposes, diverse methods, different terminology, and different settings of the studies undertaken. These differences are highlighted in Additional file 1: Table S1. The literature was also characterised by variation in the methodological rigour underpinning the studies analysed.

These features of the available literature were limitations in the context of our research objective. One reason that a definitive set of core services was not evident from the literature was because of the idiosyncratic nature with which different authors defined different sets of core services using different terminology with different methods and for different purposes. Also, the purpose of defining core services was not always explicit. Reasons for defining core services varied enormously, and included: documenting lessons learnt from case studies of successful services; to form a basis for discussion of workforce, education, and training needs; for service planning, monitoring, and evaluation; to document services mandated by legislation; planning and advocacy; maximising cost-effectiveness, and; defining the responsibilities of resident and visiting teams. Thus, the starting point for any initiative that seeks to develop a set of core services is to agree on an explicit purpose. This may vary depending on the intended audience, be they policymakers, consumers, researchers, practitioners, or health service planners. Another limitation of the literature in the context of this study was that different purposes resulted in different methods used. Thus, in future, explicitly defining a specific purpose will also provide a guide as to an appropriate method. Possible methods include: literature reviews; consultations and expert opinion; consensus methods; empirical methods; and combinations of these. Whatever method adopted, the critical factor is the need to ensure the validity and reliability of the method in relation to the stated purpose.

In managing these characteristics of the literature which, in the context of this study, were exposed as limitations, the study was able to produce a creative and perhaps more useful solution to the difficult problem of defining “core” services. Despite the considerable variability in the results from the diverse studies we encountered, we were able to synthesise common PHC dimensions across the 19 papers and the demarcations within each of these dimensions (Table 4).

In relation to context and scope, available evidence about “core” services related to widely different geographical, demographic, and epidemiological environments. These included: both developed and developing countries; regional, state, national, and global reach; as well as for specific population groups such as Indigenous populations. The dimensions and demarcations summarised in Table 4 provide an excellent starting point or checklist with which to consider the appropriate response for any given context. For example, a life span approach may be useful in a population with high needs in children and older people that are to be prioritised. This framework thus provides a valuable platform for health service providers to use in their decisions relating to how best to meet the PHC needs of their jurisdictions.

Most importantly, the critical task is to ensure that all residents (regardless of where they live) should be able to readily access the “core” services, however defined, in times of need. Recognising that many rural and remote communities cannot depend on market forces to deliver these services equitably or on the basis of need, it is clear that the way in which access to these “core” PHC services is realised will also vary from context to context. For example, some services (such as emergency retrieval and evacuation) must be available *in situ*, while others may need to be accessed by alternative models of delivery such as visiting services provided through a “hub-and-spoke” arrangement, fly in/fly out services, or telehealth. Additionally, the need to move people to services rather than services to people may require significant patient-assisted travel schemes. Any consideration of *what* services should be provided must also be accompanied by strategic thinking about *how* the services may be provided most efficiently and effectively. Past research undertaken describes a number of different models ranging from fixed services, through visiting services, and telehealth [25,26]. Arguably, not all services must be provided *in situ*. Unfortunately, there exist few rigorous evaluations of health service models comparing the cost efficiency and health effectiveness of different rural and remote health care services to help guide decisions about which services should be provided locally and which services can be just as effective in meeting health care needs through different modes of access.

Overarching this key decision about “how” to provide “which” services is the need to recognise the fiscal constraints within which every health service and funding authority operates. Financial resources are not ubiquitously available in unlimited supply, and a delimitation of the package or suite of “core” PHC services in a given context could assist decision-making in this regard. Hence the quanta of services and the provision model need to be prioritised according to community needs and context, and matched against the availability of financial resources. Furthermore, in order to ensure relevance and sustainability of service provision, it will be important for PHC health services to be responsive to changes in community needs that result from changing demography, population mobility, and ageing. Using the matrix of PHC dimensions and demarcations outlined in this paper which is based on the best available evidence, the issue of what services and how they might be accessed most effectively and efficiently can now be explored more systematically.

## Conclusion

A comprehensive review of the international literature has shown that no one agreed set of primary health care “core” services is available. Alternatively, this study has been able to synthesise the extant literature to formulate a matrix of dimensions and demarcations that will enable health authorities to assess local health needs and plan an appropriate set of core services accordingly. Just as the provision of universal access to health care through the ^a^Medicare insurance scheme has become accepted and entrenched as a right in this country, so too do Australians living in rural and remote areas have a right to expect access to a range of agreed PHC services. This systematic review provides significant guidance for the development of a set of core PHC services that can be used by health service planners to influence policy-makers and consult with consumers to guide the equitable provision of PHC. The process by which a set of core services is formulated and agreed should be evidence-based, transparent, and fit-for-purpose. The underlying methods should be explicit and rigorous. The task is for policy makers, practitioners, consumers, and researchers to work together to determine these core services for rural and remote areas of Australia and other countries with similar rural populations, and how they can be made affordable and accessible so that all citizens might enjoy a more equitable health system.

## Endnote

^a^Medicare is the publicly funded universal medical insurance scheme in Australia.

## Competing interests

The authors have no competing interests in the conduct of this systematic review or the preparation of this manuscript.

## Authors’ contributions

TAC conducted the data base searches, participated in reviewing the papers, and prepared the first draft of the manuscript. JW conceptualised the review with JSH, reviewed the initial list of titles and abstracts, participated in the review of the final set of papers, and developed and proof-read the manuscript. JSH guided and mentored the review process, formed the concept of the review with JW, and shaped the manuscript into its final form. PB and ML participated in the review of the final set of papers and contributed to the development of the manuscript. All authors read and approved the final manuscript.

## Pre-publication history

The pre-publication history for this paper can be accessed here:

http://www.biomedcentral.com/1472-6963/13/178/prepub

## Supplementary Material

Additional file 1: Table S1 Descriptions of the final set of papers in terms of the variability of different purposes, methods, terminology, and settings. Click here for file
