# Supplementary material for: What primary health care services should residents of rural and remote Australia be able to access? A systematic review of “core” primary health care services
Source: BMC Health Serv Res. 2013 May 17;13:178. doi: 10.1186/1472-6963-13-178 (PMC3663724; doi:10.1186/1472-6963-13-178)
Supplement: Additional file 1: Table S1 — Descriptions of the final set of papers in terms of the variability of different purposes, methods, terminology, and settings. [file 1472-6963-13-178-S1.docx]

Additional file 1: Table S1. *Descriptions of the final set of papers in terms of the variability of different purposes, methods, terminology, and settings.*

| **Reference** | **Core Services Specified** | **Context** | **Purpose** | **Methodology** |
| --- | --- | --- | --- | --- |
| Asuzu MC, Ogundeji MO [5] | 1. Health education and community mobilization 2. Maternal and child care including family planning 3. Promotion of food and proper nutrition 4. Water and sanitation 5. Provision of essential drugs 6. Treatment of common minor ailments and injuries 7. Prevention and control of locally endemic communicable and non-communicable diseases 8. Oral health 9. Mental health 10. PHC management information system | Nigeria | - Advocacy, supervision, monitoring, and evaluation (p. 3) - Planning and development of PHC services | - 2 consultants reviewed the literature and held meetings and key informant group discussions |
| Australian Primary Health Care Research Institute (APHCRI) [6] | 1. health promotion 2. illness prevention 3. care of the sick 4. advocacy 5. community development | Australia | - Definition of Primary Health Care | - On APHCRI website |
| Bartlett B, Boffa J [7] | 1. Clinical services 2. Social preventive programs 3. PHC Support 4. Advocacy | Australia ACCHS | - Descriptive case study, advocacy, and planning | - Historical summary of the evolution of ACCHSs (and specifically Congress) and then a case study/description of the PHC services provided by Congress which fit within a previously developed “core functions of PHC” framework |
| Bobadilla J-L, Cowley P, Musgrove P, Saxenian H [8] | 1. Public health    1. Expanded programme of immunization plus    2. School health programme    3. Tobacco and alcohol control programme    4. AIDS prevention programme    5. Other public health interventions 2. Clinical services    1. Chemotherapy against tuberculosis    2. Integrated management of the sick child    3. Family planning    4. STD treatment    5. Prenatal and delivery care    6. Limited care | Low and Middle Income Countries | - As per World Bank – essential services based on size of the burden caused by the disease risk factor, etc and cost-effectiveness of the intervention | - No country in the world can provide health services to meet all the possible needs of the population, so it is advisable to establish criteria for which services to provide. Two basic criteria are the size of the disease burden caused by a particular disease, injury or risk factor, and the cost-effectiveness of interventions to deal with it. - Analysis of cost-effectiveness of interventions which address greatest contributors to BoD - This paper is based on the World Bank's report which proposes a minimum package based on both problems and interventions: the services included are highly cost effective and deal with major threats to health |
| Brener ND, Wheeler L, Wolfe LC, Vernon-Smiley M, Caldart-Olson L [9] | 1. Standard health service (32 services listed) such as:    1. Administration of fluoride rinses    2. Administration of sport physicals    3. Crisis intervention for personal problems    4. Eating disorders treatment    5. Immunizations    6. Prenatal care referrals    7. Stress management    8. Services for gay, lesbian, or bisexual students    9. Injury prevention and safety counseling    10. STD prevention    11. Suicide prevention 2. Prevention service in 1-on-1 or small-group session (10 services listed) such as:    1. HIV prevention    2. Pregnancy prevention    3. Violence prevention    4. Nutrition and dietary behavior counselling | Schools in the US | - Defining school health activities | - Empirical data relating to current practice. Not really theory-driven; underlying education policy document cited. - The Centers for Disease Control and Prevention conducts the School Health Policies and Programs Study (SHPPS) every 6 years. In 2006 computer-assisted telephone interviews or self-administered mail questionnaires were completed. The data obtained from these methods were used in this paper. |
| CRH [10] | 1. Antenatal care & childbirth 2. Postnatal care 3. Young child health 4. Older child & youth health 5. Oral & dental health 6. Environmental health 7. Immunisation 8. Communicable disease control 9. Well men and women services 10. Chronic disease management 11. Mental health 12. Primary disability services for children & adults 13. Aged care 14. Palliative care 15. Acute care services 16. Emergency medical consultation & evacuation 17. Hospital liaison, discharge & patient travel 18. Pharmacy services 19. Alcohol & other drugs 20. Child protection 21. Sexual health services 22. Sexual assault 23. Domestic & family violence | Remote Northern Territory, Australia | - Define and evaluate the core services, planning, defining resident team and visiting team, define measures for monitoring, and evaluation | - Audit of DHF documents, meeting with key people, refining and development of successive drafts, validation by Steering Committee of final draft |
| Farrow S, Harrison W, Kaluzny A, Newsome K [11] | The core activities formed 3 clusters: patient examination, laboratory procedures, treatment procedures  Core activities:   1. Therapeutic injection 2. Ear irrigation 3. Prescribing medication 4. Taking patient’s history 5. Examination of the soft tissues of the mouth 6. Dressing a burn or wound 7. Lab blood test for hematology 8. Pap smear 9. Urinalysis 10. Pelvic examination 11. Immunization 12. Physical examination for entering school 13. Pregnancy testing 14. TB skin test 15. Routine obstetrics care 16. Patient counseling 17. Well baby care 18. Urine culture 19. Microscopic examination of urine 20. Breast examination as screening procedure 21. Testing vision 22. Suture removal 23. Taking blood pressure as screening procedure 24. Teaching diabetic patient to inject insulin 25. Lab blood test for chemistry 26. Dietary counseling 27. Simple diagnostic x-ray 28. Sickle cell test as screening procedure 29. Family planning counseling 30. Eye examination as screening for glaucoma 31. IUD insertion | North Carolina, US | - Defining services in order to “planning the evaluation efforts that make different groups of providers …” - Defining a method that can be repeated to help understand intergroup and regional differences ie Method Improvement | - An activity-based definition of primary care was derived from ratings of 59 representative health care activities by a group of state health administrators, a group of local health department directors, and a random sample of primary care physicians practicing in North Carolina - The justification for taking mean ratings of 4.0 or greater seemed to be that this corresponded “to the two most definitely primary care scale points” (p. 96) - The list of activities was developed by reviewing documents and consulting with key people |
| AH&MRC [12] | 1. Medical Care    1. Clinical Health Services    2. Pharmaceutical services    3. Preventative Care    4. Medical Records & Health Information System 2. Dental Services    1. Dental clinical services    2. Preventative dental care    3. Dental Records & Information System 3. Health Related Services and Community Support Services    1. Social and emotional wellbeing services    2. Psychiatric services and care    3. Aged care services    4. Podiatry services    5. Ophthalmology services    6. School based activities    7. Funeral assistance    8. Prison advocacy services    9. Needle exchange services    10. Audiology services | ACCHS, NSW | - To reflect the Aboriginal definition of holistic health | - Not specified. States on p. 7 “The following list of core services are those which are provided, subject to adequate funding, in many ACCHS’s and reflect the Aboriginal definition of holistic health” |
| OATSIH [13] | Described as the service elements of comprehensive primary health care.   1. Clinical care    1. Treatment of acute illness    2. Emergency care    3. Management of chronic conditions 2. Population health programs such as:    1. Immunization    2. Antenatal care    3. Screening    4. Specific health promotion programs 3. Facilitation of access to secondary and tertiary health services 4. Client/community assistance and advocacy on health-related matters within the health and non-health sectors | ATSI population Australia | - Descriptive account of Indigenous comprehensive PHC and lessons learnt from case studies of successful services and programs | - Uses Alma-Ata definition of primary health care - Doesn’t specify methods for delineating the core elements it proposes |
| Lyle D, Kerr C [14] | 1. 24 hour emergency care 2. Immunization 3. A specific program for child health 4. Antenatal care 5. A prevention and control program for sexually transmissible and HIV infections 6. Referral and evaluation system 7. Chronic disease surveillance and treatment 8. Health worker training and support programs 9. Systematic approaches to staff recruitment, orientation, support and career development 10. Data collection on population, interventions and outcomes 11. Evaluation of activities 12. Targeted and evaluated programs to manage, reduce and prevent substance abuse | Remote Australia | - As a basis for discussing workforce education and training issues | - Provides a reference and says that these are “what several experienced health professionals in remote areas regard as a set of core activities that are required for the delivery of comprehensive primary health care services.” (p. 159). |
| McDonald MC [15] | 1. Environmental health 2. Primary clinical care 3. Diabetes control 4. Alcohol and drugs 5. Child health 6. Mental health 7. Communicable disease control (CDC) 8. Oral health 9. Women’s health 10. Post acute care 11. Extended care 12. Rehabilitation 13. Discharge planning and respite services 14. Sexual health 15. Allied health 16. Family health | Remote Indigenous Australia | - For service planning and monitoring purposes | - No method referred to - No body of knowledge specified - Justification given for list of services on p.46: The services are based on documented Sector priorities and what is already available. Once a final list has been developed through appropriate consultative processes, it should be the standard for maintaining a consistent level of quality and content for Primary Health Care services in the sector. [doesn’t provide a reference for the ‘documented Sector priorities’] |
| NACCHO [16] | 1. Medical Care    1. Clinical Health Services    2. Pharmaceutical services    3. Preventative Care    4. Medical Records & Health Information System 2. Dental Services    1. Dental clinical services    2. Preventative dental care    3. Dental Records & Information System 3. Health Related Services and Community Support Services    1. Social and emotional wellbeing services    2. Psychiatric services and care    3. Aged care services    4. Podiatry services    5. Ophthalmology services    6. School based activities    7. Funeral assistance    8. Prison advocacy services    9. Needle exchange services    10. Audiology services | Aboriginal Community Controlled Health Services | - Reflects in the NACCHO constitution the services provided by ACCHOs to reflect the “holistic” nature of health | - Methods not specified - Use a definition of primary health which is stated (on p. 54) as adapted from the Alma-Ata definition - States on p. 55: “The following list of core services are those which are provided, subject to adequate funding, in many ACCHS’s and reflect the Aboriginal definition of holistic health” |
| NATSIHC [17] | Define the elements of a comprehensive primary health care service including at least:   1. Clinical services 2. Policy and program management 3. Substance misuse 4. Sexual health services 5. Mental health 6. Community development and population health programs, including a focus on prevention including nutrition and lifestyle risk factors | ATSI Australia | - As per NACCHO document | - Doesn’t specify methods - Based on *1989 National Aboriginal Health Strategy* and on the implementation and partnerships that have developed since 1989. This *Consultation Draft* has been developed under the auspices of the National Aboriginal and Torres Strait Islander Health Council - Doesn’t really review the literature but cover a broad range of literature including previous policy documents to support its position |
| Scrimgeour D [18] | Canada:  ‘Mandatory programs’ specified under the Health Transfer policy:   1. Communicable disease control 2. Environmental/occ health & safety 3. Treatment services 4. Emergency response plan (p 69)   Remote Australia (pp 106-111)- detailed description of cores services and the process of implementation. Six core service components:   1. Primary clinical care 2. Population health/preventive care 3. Specialist & ancillary services 4. Support systems 5. Special program 6. Advocacy & policy development   Health promotion should be integrated within each of these components.  1-4 are ‘core medical services’ delivered by any provider at community level & determined by a ‘core medical services committee’ at a regional level.  Special programs: communities should be funded to develop these in areas of need which they identify. | First Nations, Canada; Remote Indigenous Australia | - Description of “Mandatory programs” as synthesised by government policy; planning and advocacy | - Literature reviewed to develop a list and typology of core services. Methodological information about core services is not explicit. |
| Scrimgeour D [19] | Canada:   1. ‘Mandatory programs’ specified under the Health Transfer policy:  - Communicable disease control - Environmental/occ health & safety - Treatment services - Emergency response plan (p 8)  1. List of services at Montreal Lake (a comm. Controlled health service):  - Comm disease control - Primary health care including assessment, diagnosis & treatment, emergency treatment - School health - Community environmental health - Adult health, incl assessment, monitoring & treatment of chronic disease - Mental health services - Multiproblem family support services - Social health activities - Diabetic education - Traditional health education - Elder health services - Alcohol & drug abuse program - Weekly physician service - Monthly dental service (pp 17-18)  1. List of ‘second level services’ in the Prince Albert Grand Council Transfer Agreement. These appear to be centralized services made available to a range of services within the region.  - Health directors’ working group - Consulting services including:   - Addictions   - Health promotion   - Mental health   - Research & epidemiology   - Community health development   - Nursing supervisions   - Medical health officer   - Environmental health officer   - Dental therapy - Capital management & planning - A ‘medical boarding house’ and a residential drug & alcohol treatment centre - Finance & administration - Liaison & management (pp 19-20)   There is also a strong focus on traditional medicine and traditional healers within the PHC services.  New Zealand:  The author describes the services provided by one nurse-led community organisation (pp 39-40). These include child health, adolescent health, adult health, health promotion & education programs. | First Nations, Canada; New Zealand Maori | - Presented programs under agreed government policy; - Descriptive case study | - A number of descriptive case studies |
| Tilton E, Thomas D [20] | Core Functions of primary health care:   1. Clinical services    1. Treatment    2. Prevention & early intervention    3. Rehabilitation and recovery    4. Clinical support systems 2. Health promotion    1. Building health public policy    2. Creating supportive environments    3. Supporting community action and development    4. Health information, education and skills development    5. Orienting health services towards health promotion    6. Evidence and evaluation in health promotion 3. Corporate services & infrastructure    1. Management and leadership    2. Workforce and HR management    3. Staff development, training and education    4. Financial management    5. Administrative, legal & other services    6. Infrastructure and infrastructure management    7. Information technology    8. Quality systems 4. Advocacy, knowledge & research, policy & planning    1. Advocacy    2. Knowledge and research    3. Policy and planning 5. Community engagement, control & cultural safety    1. Engaging individual clients with their health and care    2. Supporting community participation    3. Governance and community control    4. Cultural safety | NT Aboriginal population | - For planning purposes and “to direct and underpin decisions about health service structures and delivery” (p. 1) - Policy document - Instrument for planning - Planning and evaluation tool (p. 3) | - A process of consultation and liaison with key stakeholders and informants using advisory groups for advice and guidance also |
| WACHS [21] | 1. Antenatal and Maternity Care 2. Child and School Health Service 3. Youth Health Service 4. Well Men and Well Women Services 5. Mental Health Services 6. Alcohol and Drug Services 7. Oral and Dental Health Services 8. Chronic Disease Care Coordination 9. Palliative Care 10. Healthy Ageing and Aged Care 11. Public Health Services | Remote Western Australia | - Not explicit but part of policy document and to be used as a basis to plan service procedures, models and strategies | - States on p. 22: “From analysis of available data, information from other jurisdictions including Northern Territory, Queensland, Canada and Alaska and New Zealand existing planning processes and local knowledge the following have been identified as primary health care Core Service / Program areas for the Kimberley |
| World Bank [22] | 1. Public health    1. Immunizations    2. School-based health services    3. Information and selected services for family planning and nutrition    4. Programs to reduce tobacco and alcohol consumption    5. Regulatory action, information, and limited public investments to improve the household environment    6. AIDS prevention 2. Essential clinical services    1. Services to ensure pregnancy-related (prenatal, childbirth, and postpartum) care    2. Family planning services    3. Tuberculosis control    4. Control of STDs    5. Care for the common serious illnesses of young children – diarrheal disease, acute respiratory infection, measles, malaria, and acute malnutrition | Lower and Middle Income countries | - Purchase of essential services based on size of the burden caused by the disease risk factor, etc and cost-effectiveness of the intervention | - a minimum package based on both problems and interventions: the services included are highly cost effective and deal with major threats to health |
| WHO [23] | Document says that primary health care includes at least:   1. education concerning prevailing health problems and the methods of preventing and controlling them 2. promotion of food supply and proper nutrition 3. an adequate supply of safe water and basic sanitation 4. maternal and child health care, including family planning 5. immunization against the major infectious diseases 6. prevention and control of locally endemic diseases 7. appropriate treatment of common diseases and injuries 8. provision of essential drugs | Global | - Engendering political commitment to basic elimination of health inequity | - Doesn’t specify how it arrived at these but listed them as a declaration by the conference attendees. - Defines primary health care as “Primary health care is essential health care based on practical, scientifically sound and socially acceptable methods and technology made universally accessible to individuals and families in the community through their full participation and at a cost that the community and country can afford to maintain at every stage of their development in the spirit of self reliance and self-determination. It forms an integral part both of the country's health system, of which it is the central function and main focus, and of the overall social and economic development of the community. It is the first level of contact of individuals, the family and community with the national health system bringing health care as close as possible to where people live and work, and constitutes the first element of a continuing health care process.” (pp. 1-2) |
